# Magnetic Sector Secondary Ion Mass Spectrometry on FIB-SEM Instruments for Nanoscale Chemical Imaging

**DOI:** 10.1021/acs.analchem.2c01410

**Published:** 2022-07-21

**Authors:** Olivier De Castro, Jean-Nicolas Audinot, Hung Quang Hoang, Chérif Coulbary, Olivier Bouton, Rachid Barrahma, Alexander Ost, Charlotte Stoffels, Chengge Jiao, Mikhail Dutka, Michal Geryk, Tom Wirtz

**Affiliations:** † Advanced Instrumentation for Nano-Analytics, MRT Department, Luxembourg Institute of Science and Technology, 41 rue du Brill, L-4422 Belvaux, Luxembourg; ‡ Prototyping, MRT Department, Luxembourg Institute of Science and Technology, 41 rue du Brill, L-4422 Belvaux, Luxembourg; § Faculty of Science, Technology and Medicine, University of Luxembourg, 2 Avenue de l’Université, L-4365 Esch-sur-Alzette, Luxembourg; ∥ Thermo Fisher Scientific; Achtseweg Noord 5, 5651 GG Eindhoven, Netherlands; ⊥ Thermo Fisher Scientific; Vlastimila Pecha 12, 627 00 Brno, Czech Republic

## Abstract

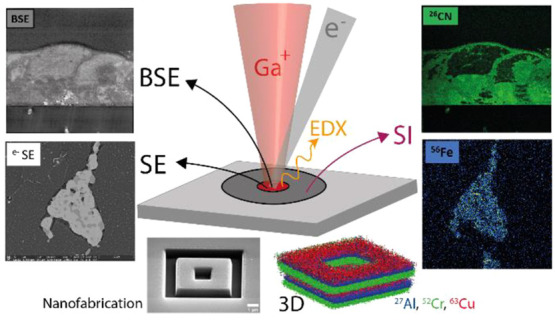

The structural, morphological, and chemical characterization of samples is of utmost importance for a large number of scientific fields. Furthermore, this characterization very often needs to be performed in three dimensions and at length scales down to the nanometer. Therefore, there is a stringent necessity to develop appropriate instrumentational solutions to fulfill these needs. Here we report on the deployment of magnetic sector secondary ion mass spectrometry (SIMS) on a type of instrument widely used for such nanoscale investigations, namely, focused ion beam (FIB)–scanning electron microscopy (SEM) instruments. First, we present the layout of the FIB-SEM-SIMS instrument and address its performance by using specific test samples. The achieved performance can be summarized as follows: an overall secondary ion beam transmission above 40%, a mass resolving power (*M*/Δ*M*) of more than 400, a detectable mass range from 1 to 400 amu, a lateral resolution in two-dimensional (2D) chemical imaging mode of 15 nm, and a depth resolution of ∼4 nm at 3.0 keV of beam landing energy. Second, we show results (depth profiling, 2D imaging, three-dimensional imaging) obtained in a wide range of areas, such as battery research, photovoltaics, multilayered samples, and life science applications. We hereby highlight the system’s versatile capability of conducting high-performance correlative studies in the fields of materials science and life sciences.

A common fact about a large number of technological and scientific fields is that, in order to answer emerging critical questions, new and innovative characterization tools need to be developed. An essential element of this framework consists of combining structural and chemical information at the nanometer scale. The application areas of such characterization instruments span a wide range of fields and include the imaging of features in highly complex electronic devices at high lateral resolution (e.g., dopant distributions, grain structures, and boundaries) as well as the precise recording of subcellular chemical information in biological specimens to obtain better knowledge of the ongoing processes at the physiological level. Key characteristics that are required are highest spatial resolution, excellent chemical sensitivity, high dynamic range (for the detection and mapping of elemental concentrations varying over several orders of magnitude), and isotopic selectivity.

One particular type of instrument, based on the combination of a high-resolution electron beam column (with spot sizes in the nanometer range) and a focused ion beam (FIB) column,^1−3^ is being used extensively in this context. The main benefit of this is that the user has the ability to perform secondary electron microscopy (SEM) imaging in combination with FIB sample preparation and nanofabrication tasks in a single tool. Such instruments are typically referred to as FIB-SEM or dual-beam instruments.

The well-established technique for compositional analysis deployed on such instruments is energy-dispersive X-ray spectroscopy (EDX).^4−6^ Nevertheless, EDX is not able to distinguish between isotopes or detect trace elements (poor detection limit of ∼1000 ppm^7^). Furthermore, in the low Z-element range (below boron), the detection of hydrogen is impossible, and the detection of Li very challenging.^8,9^

An analytical technique offering higher sensitivity (detection limits down to the ppm range) is secondary ion mass spectrometry (SIMS), which also enables the detection of all elements from H to U while differentiating between isotopes and offering a high dynamic range.^10,11^ The effort to offer SIMS capability on single-column FIB instruments goes back to the 1980s, as demonstrated by the works of Levi-Setti et al.,^12,13^ and has been extended in more recent years to FIB-SEM instruments.

One option to perform SIMS on FIB-SEM instruments is based on the use of a time-of-flight (TOF) spectrometer as the SIMS spectrometer.^14^ More specifically, such a setup is often based on an orthogonal TOF (o-TOF) configuration in which the secondary ion (SI) beam is pulsed while working with a continuous primary ion beam.^15^ While such TOF systems have the benefit of being compact in size and having quasi-parallel mass detection, they suffer from a low duty cycle (while the sample is constantly eroded by the DC beam, only fractions of the SIs are pulsed into the TOF systems), which in addition is mass-dependent. This results in a reduced overall sensitivity, which also varies as a square root function with respect to the mass.^15^

Another type of SIMS spectrometer being used on FIB-based instruments is quadrupole spectrometers.^16^ Chater et al. reported a single-column FIB instrument equipped with two quadrupole spectrometers, to detect both positive and negative SIs at the same time.^17^ Nevertheless, these systems work in serial acquisition (meaning that ions of different masses are detected sequentially rather than simultaneously) and are not suitable for fast chemical imaging.

A third kind of SIMS spectrometer is magnetic sector-based spectrometers. These operate in DC mode (no pulsing and hence no losses due to duty cycles) and have high overall transmission of SIs, conferring on them important advantages in terms of sensitivity and speed over TOF spectrometers. Furthermore, in contrast to quadrupole spectrometers, they offer parallel mass detection and are hence optimized for fast imaging tasks. This function is specifically achieved in a Mattauch-Herzog configuration, which is characterized by an elongated straight focal plane covering the full mass range of the spectrometer.^18^ Finally, high mass resolution can be achieved when magnetic sector SIMS systems are operating in double-focusing condition. While magnetic sector SIMS systems used to be bulky and heavy, modern systems can be compact and considerably lighter thanks to advances in charged particle optics, electromagnet coil concepts, and mechanical assembly design.^19,20^

We report here on an FIB-SEM instrument incorporating a newly developed compact double-focusing magnetic sector SIMS spectrometer. The instrument offers key capabilities, such as (1) nanoscale lateral resolution imaging in SEM mode; (2) nanoscale, high sensitivity, and high dynamic range chemical/elemental analysis in SIMS mode, including sub-20 nm imaging resolution and sub-5 nm depth resolution; (3) isotopic analysis; (4) correlation of SIMS data with other data sets, such as secondary electron (SE) images, backscattered electron (BSE) data, and EDX data. The SIMS system can be operated in several modes: recording of mass spectra, two-dimensional (2D) chemical imaging, three-dimensional (3D) chemical imaging by recording, and stacking the multiple 2D chemical maps obtained when removing the sample material layer-by-layer, and depth profiling (following selected mass signals while milling the sample). All these capabilities pave the way for a large number of application possibilities, ranging from analytics needing high sensitivity, SIMS imaging at highest lateral resolution (15–20 nm), and FIB nanofabrication (milling/patterning) with in situ process control, to multimodal approaches correlating SIMS data with SE, BSE, and EDX data obtained from the same instrument.

## Instrument Design

**Figure 1:**
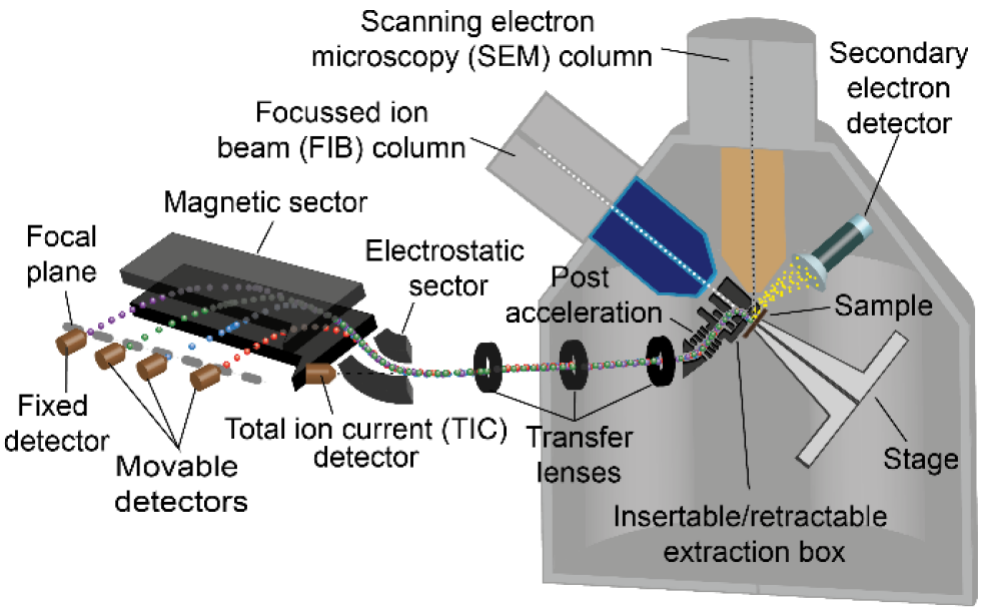
Schematic layout of the FIB-SEM-SIMS instrument.

The instrument described here is based on a commercially available high-vacuum FIB-SEM platform called Scios from Thermo Fisher Scientific.^21,22^ As shown in Figure 1, the instrument is equipped with a vertically orientated SEM column based on a field emission source. The SEM-column allows electron beam landing (impact) energies from 200 eV to 30 keV, offering a lateral resolution of 1–2 nm at 1 keV. The instrument is equipped with various subsystems for electron beam-induced SE signal detection as well as for BSE signal detection. A Ga liquid metal ion source (LMIS)-based FIB column (Ga-FIB) is installed at 52° with respect to the vertical SEM axis. The Ga-FIB offers an ion beam landing energy range from 500 eV to 30 keV, with probe currents spanning from 1.5 pA to 65 nA. The Ga^+^ primary beam can be used for ion beam-induced SE imaging as well as for ion beam milling/fabrication and analytical purposes, including SIMS. The smallest probe size of 3 nm is achievable at 30 keV. Optionally, specific gas injection systems (GIS) can be installed to offer, for example, the in situ platinum coating of sample surfaces or injection of precursors for other fabrication tasks. The eucentric beam coincidence point of the instrument is located at the FIB working distance (WD) of 19 mm and the SEM WD of 7 mm. When one positions the region of interest (ROI) of the sample at this precise location, it is possible to perform sample (stage) tilt movements without any lateral displacements of the ROI under either primary beam. This allows one to do nanofabrication with the ion beam while performing quasi-simultaneous SEM imaging of the exact same ROI.

To perform SIMS on the Scios instrument, we developed a dedicated compact magnetic sector SIMS spectrometer that can be installed as an add-on attachment, allowing us to do an SIMS analysis with the Ga-FIB while not interfering with or reducing the performance of the standard SEM and FIB operations. Because of the nature of the dual-beam operation, there are several challenges in the development and integration of such a dedicated SIMS system, including, in particular, the design of the extraction and transfer optics. First, the SIMS extraction optics need to be integrated into an extremely limited space between the SEM column, the FIB column, and the sample while not disturbing the operation of other detectors (i.e., the SE detector). Second, the SIMS needs to be operated with the sample at the eucentric beam coincidence point, allowing us to perform both SIMS and SEM analyses of the same ROI. Third, the integration of the SIMS optics needs to be compatible with available existing ports on the Scios and the orientation of the FIB column. Finally, apart from the hardware integration challenges mentioned above, the SIMS system should not degrade the probe size of the FIB column in the SIMS mode beyond the dimensions of the collision cascades triggered within the sample upon ion impacts to enable nanoscale resolution. Moreover, it should have a high extraction efficiency to maintain high sensitivity even when working with nanoscale sputtered voxels (from all sputtered particles only ∼1% leave the sample as SIs, but this fraction significantly depends on the considered element and the sample composition (matrix effect)). The choice of the primary ion species has an influence on the SI signal and hence on the sensitivity. The relatively high mass of Ga (as compared to light species such as He or Ne used on the helium ion microscope) results in high sputter yields and therefore also increases the SI signals. In combination with the residual oxygen in the analysis chamber when working at a typical pressure of 10^–7^ mbar, Ga also allows for good ionization yields in the positive SI mode. The yields can be further increased by increasing the partial oxygen pressure in the chamber via an O_2_ leak. For negative SI detection, the use of very electropositive primary ions such as Cs would lead to even higher ionization yields.^19^

**Figure 2:**
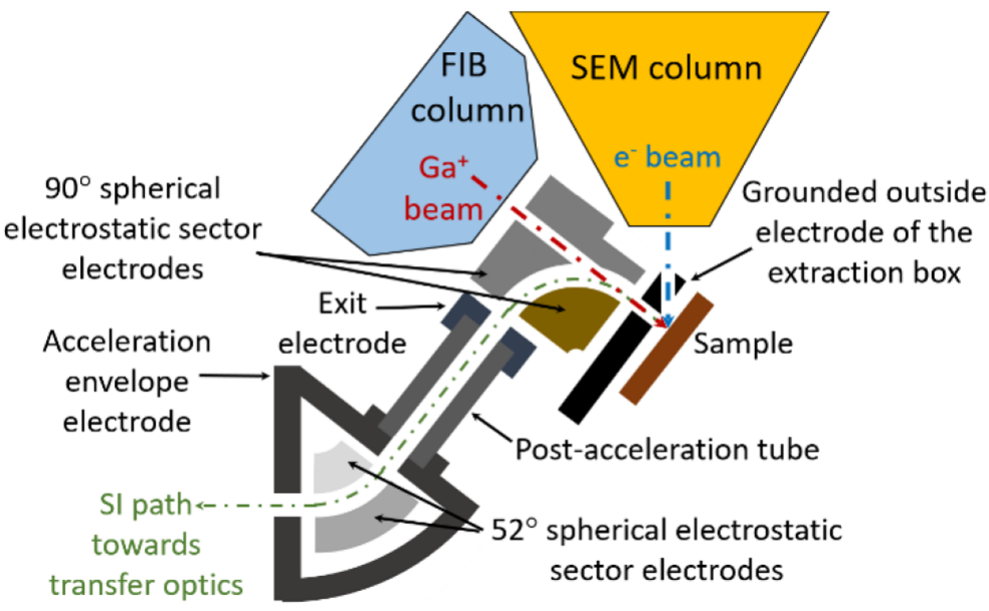
Schematic layout of the extraction box region close to the sample/column end nozzle area, post acceleration region, and second 52° bending electrostatic sector.

Addressing all of the above challenges, a dedicated SIMS system, as schematically presented in Figure 1, was developed to integrate it into the Scios (a photograph of the FIB-SEM-SIMS instrument is shown in Figure S1 of the Supporting Information). To maintain a high extraction efficiency as well as to position the sample at the eucentric point during the SIMS operation, the ion extraction optics are based on an extraction box, which was described elsewhere.^19^ During SIMS operation, the extraction box is placed in between the sample and the nose cone of the FIB column, allowing both the normal incident angle of the primary beam on the sample and the normal extraction of the SIs from the sample. Furthermore, this chosen geometry allows the SIMS operation with the sample at the eucentric point independently of the strength of the extraction field (or sample bias). As shown schematically in Figure 2, this extraction box has to be carefully tailored to fit into the extremely limited space between the FIB column and the SEM column. The overall height of the extraction box is 16.5 mm. Note that the design also allows the SEM operation to be used through an aperture of 0.5 mm in diameter integrated at 52° with respect to the FIB column axis in the outer grounded electrode of the extraction box.

During the SIMS operation, the extraction box is set at 0.5 mm above the sample, and the sample is biased to ±500 V for positive and negative SI extraction, respectively. The SIs are extracted by the electric field created by the sample bias potential and the grounded first electrode of the extraction box. They are then further transported by a 90° spherical electrostatic sector. As the orientation of the FIB column is at 52° with respect to the vertical axis, these transported ions have to be bent by another 52° in order to be aligned with the mass spectrometer, which is mounted in the horizontal plane on the Scios. Therefore, a second spherical electrostatic sector with a bending angle of 52°, in combination with a postacceleration tube placed between the two electrostatic sectors, is used as illustrated in Figure 2. The acceleration tube allows us to accelerate the SIs from 500 eV to 3.5 keV in order to maximize the transmission of the subsequent optics. Note that all the subsequent optics from the 52° electrostatic sector to the detector are electrically floated at ±3 kV to maintain the kinetic energy of the SIs at 3.5 keV throughout the SIMS system. A dedicated transfer optics consisting of three lenses and four quadrupole deflectors then transports the SIs further to the mass analyzer placed outside of the Scios chamber (Figure 1). When the SIMS is not used, the complete extraction and transfer optics can be retracted by means of piezo-positioners to a storage (parking) position close to the chamber wall, restoring all the normal operation capabilities of the Scios.

The mass analyzer used in this system is based on a modified Mattauch-Herzog-type magnetic sector configuration, which consists of a 60° spherical electrostatic sector and 75° magnetic sector. An electromagnet sector is used, offering both magnetic field polarities for the positive and negative SIMS modes. The pole pieces and yoke of the magnetic sector are carefully designed in order to minimize their size and volume, with the target of reducing weight while maximizing the magnetic field strength capacity. This magnetic sector, in combination with the transfer optics, allows a parallel detectable mass range (*M*_max_/*M*_min_ = 120; *M*_max_ of 400 amu at a magnetic field of 700 mT) along its focal plane of 400 mm. A mass resolving power (*M*/Δ*M*) above 400 is achieved with an overall transmission of above 40%.

The current system is equipped with four channeltron detectors. Three detectors can be moved independently by piezo drives along the focal plane. The fourth detector is fixed at the highest radius position of the focal plane. This arrangement of the detection system allows the flexibility to select four masses to be recorded for SIMS analysis in parallel. Note that this SIMS system offers the capability to integrate a continuous full range focal plane detector, allowing full parallel detection of all the masses and hyperspectral SIMS analysis. This type of focal plane detector has been developed and reported elsewhere,^23^ and it will equip the next-generation SIMS on this FIB-SEM-SIMS system. Moreover, the system integrates an additional channeltron detector positioned at the end of the transfer optics for total ion current (TIC) measurements. This option is beneficial for optics alignment purposes as well as for nonmass filtered imaging in both SIMS polarities, giving further complementary contrast imaging capabilities.

## Experimental Section

The BAM-L200 sample consists of a nanoscale stripe pattern for length calibration and specification of spatial resolution prepared from a cross-sectioned epitaxially grown layer stack of Al_*x*_Ga_1–*x*_As and In_*x*_Ga_1–*x*_As on a GaAs.^24^

For the multilayer sample, Cu, Al, and Cr were deposited successively on a silicon wafer by Plasma Vapor Deposition (PVD) (Kurt J. Lesker Company). Power at the rate of 30 W dc was applied successively on three targets of aluminum, copper, and chromium (2 in. in diameter and 1/4 in. thick with a purity of 99.95%), and to homogenize the deposit, the silicon substrate was rotated at a speed of 5 rpm. This stacking of six layers was characterized by ultralow impact energy SIMS (Sc-Ultra, Cameca) using a primary cesium ion beam of 5 nA with an impact energy of 1 keV (Supporting Information). Each layer thickness, Cu (22.6 nm)/Al (11.7 nm)/Cr (33.7 nm)/Cu (26.5 nm)/Al (13.8 nm)/Cr (26.8 nm), was determined by profilometry (Tencor P20).

The hybrid organic–inorganic halide lead perovskite absorber containing Cs cations was fabricated by the perovskite deposition of a solution by spin coating on a glass substrate. The solution was obtained with the desired proportions of the precursor solution of PbI_2_(TCI), PbI_2_ (TCI), CsPbI_3_ (TCI), formamidinium iodide (Dyesol), and methylammonium bromide (Dyesol) dissolved in a 1:4 mixture of dimethyl sulfoxide (DMSO)/dimethylformamide (DMF). The perovskite solution was spun at 5000 rpm for 30 s using a ramp of 3000 rpms^–1^. A final heating at 100 °C for 50 min was performed to complete the process of the crystallization.

As described by Schoppe et al.,^25^ the copper indium gallium selenium (CIGS) film was grown by coevaporation of the elements with a high-temperature multistage in-line process. The Rb was introduced into the absorber layer via the deposition of an RbF layer of ∼300 nm in thickness and annealing in vacuum at 355 °C for 20 min, allowing a constant Rb diffusion in the CIGS layer. Residual RbF on the CIGS surface was removed with HCl. For the FIB-SEM-SIMS analysis, a piece of 2 × 2 cm^2^ was cut with manual tools.

The Al–Li–Cu–Mg alloy sample material is prepared as described in Xu et al.^26^

Biosample: All chemicals were purchased from Sigma-Aldrich. Caco-2 cells were cultured and seeded on an insert (Millicell Hanging Inserts, PET 1 μm, 24-well plate). After 21 d of differentiation, cells were exposed to 5 μM perfluorooctanoic acid for 24 h. Cells on an insert were fixed with 5% glutaraldehyde in Dulbecco’s modified phosphate buffered saline (DPBS) for 12 h at 4 °C and postfixed with 1% osmium tetroxide in DPBS for 1 h at room temperature. Afterward, samples were dehydrated with a series of graded ethanol solution (30%; 50%; 70%; 90% for 10 min; 100% for 2 h) and embedded in Spurr resin. The resin blocks were sliced into 300 nm semithin sections (ultramicrotome, Leica) and deposited on silicon wafers (Sil’tronix). Finally, the sections were coated with a 5 nm gold film by sputtering (Leica).

## Results and Discussion

### SIMS Performance: Mass Spectrum Recording

The mass spectra are recorded while scanning the Ga^+^ beam over a surface (e.g., from 2 × 2 μm^2^ to 100 × 100 μm^2^) to average the signal intensity over the ROI. The mass spectra are generated by either scanning the magnetic field with the detectors placed at fixed positions along the focal plane or by moving at least one of the three movable detectors (i.e., detector scan mode) at a fixed magnetic field.

**Figure 3:**
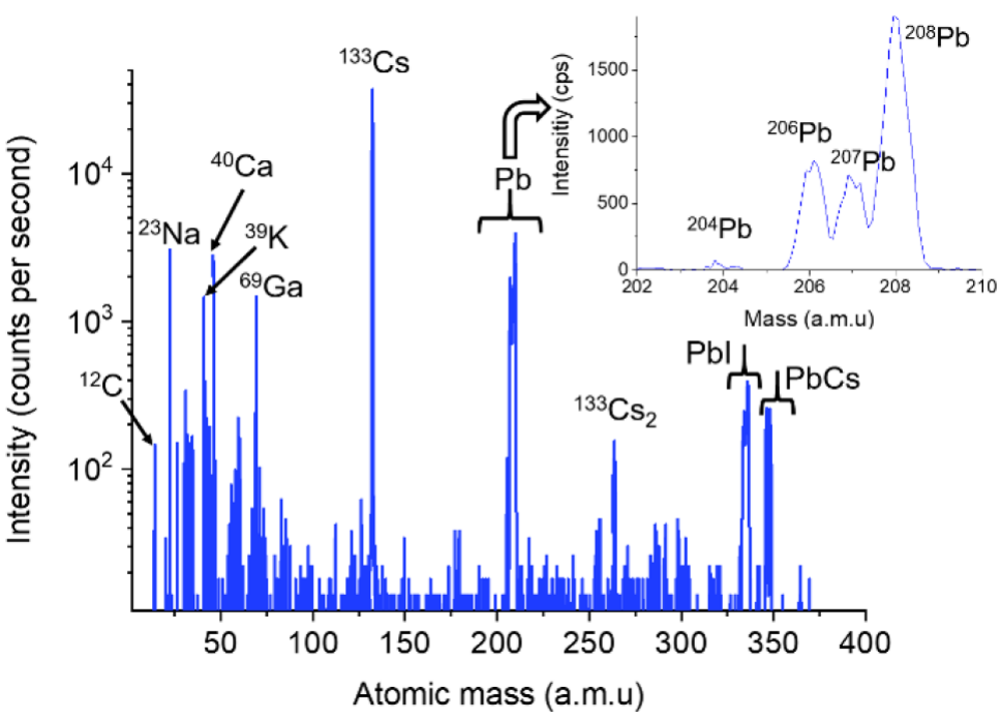
Mass spectrum (magnetic field scan of 5 to 700 mT) of a hybrid organic–inorganic halide lead perovskite absorber containing Cs cations^27^ using a Ga^+^ primary ion beam. The enlarged spectrum (upper-right) shows the distribution of the main Pb isotopes.

Figure 3 shows a mass spectrum of a hybrid organic–inorganic halide lead perovskite absorber containing Cs cations.^27^ The mass spectrum was obtained from an area of 50 × 50 μm^2^ using a 30 keV Ga^+^ beam with a current of 6 pA. The magnetic field was scanned from 5 to 700 mT in 0.10 mT steps, allowing us to cover a mass range from 0 to 380 amu (using the fixed detector). The counting time (frame dwell time) was 250 ms per step. The main elements that constitute the photovoltaic film were detected as well as some small cluster ions (such as Cs_2_, PbI, PbCs). The four lead isotopes, ^204^Pb, ^206^Pb, ^207^Pb, and ^208^Pb, were detected with an isotopic ratio close to the theoretical values (1%, 24%, 22%, and 52%, respectively). The mass resolving power (full width at half-maximum (FWHM)) *M*/Δ*M* calculated on the ^208^Pb peak amounted to 410.

### SIMS Performance: Depth Profiling

The depth-profiling mode is traditionally one of the most frequent uses of SIMS.^28^ The best depth resolution can be obtained with low-impact/beam-landing energies, typically below 1 keV.

**Figure 4:**
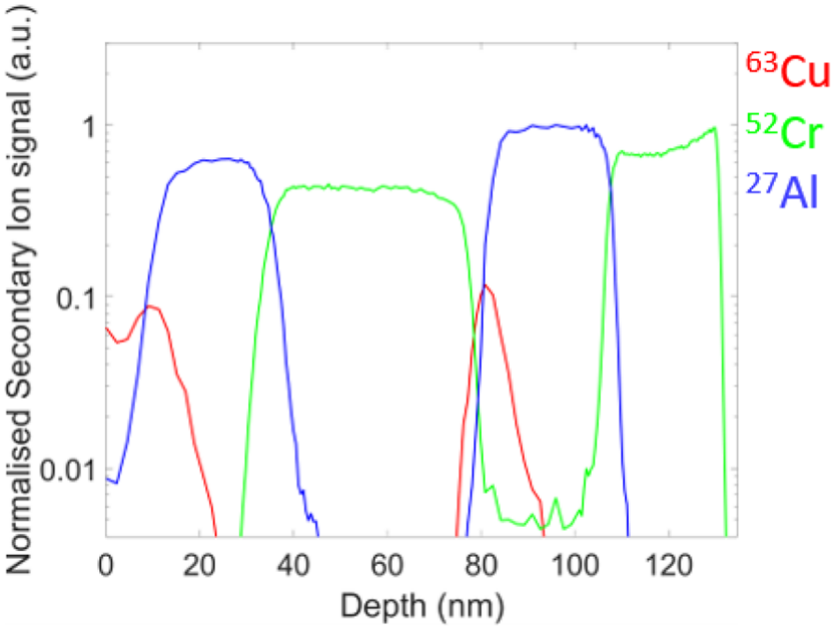
Depth profiling on a multilayer sample: Cu (23 nm)/Al (11 nm)/Cr (34 nm)/Cu (27 nm)/Al (14 nm)/Cr (27 nm)/silicon substrate. The depth profile was created by integrating the counts for each element obtained in each scanned frame (in arbitrary units).

A multilayer sample, consisting of copper, aluminum, and chromium (two layers each) was analyzed to create a depth profile from scanned frame integration (Figure 4). The SIMS detectors were aligned to measure the most abundant isotopes, that is, ^27^Al, ^52^Cr, ^63^Cu, and ^69^Ga. The gallium signal was acquired for SI signal normalization purposes. A 6 pA Ga^+^ beam with 3 keV landing energy (chosen for both good signal statistics and depth resolution) was scanned over a surface of 10 × 10 μm^2^, at 512 × 512 pixels with a dwell time of 1 ms/pixel. Figure 4 shows the depth profiles. The total counts of Cu, Al, and Cr were summed up for the scanned area and were normalized with respect to the Ga signal to compensate for any instrumental drift of the SI beam alignment, the sample stage position, or the FIB column parameters, due to the long acquisition time (in total 12 h). The time scale was converted to a depth scale (in nm) on the *x*-axis by imposing the layer thickness determined from measurements previously obtained on the same sample using a well-calibrated stand-alone SIMS instrument, the SC Ultra^29^ (Supporting Information Figure S2). A depth resolution (decay length/decade) of 3.8 nm was determined from the raw profile (average of rising and falling edge for each layer).

### SIMS Performance: 2D Imaging

**Figure 5:**
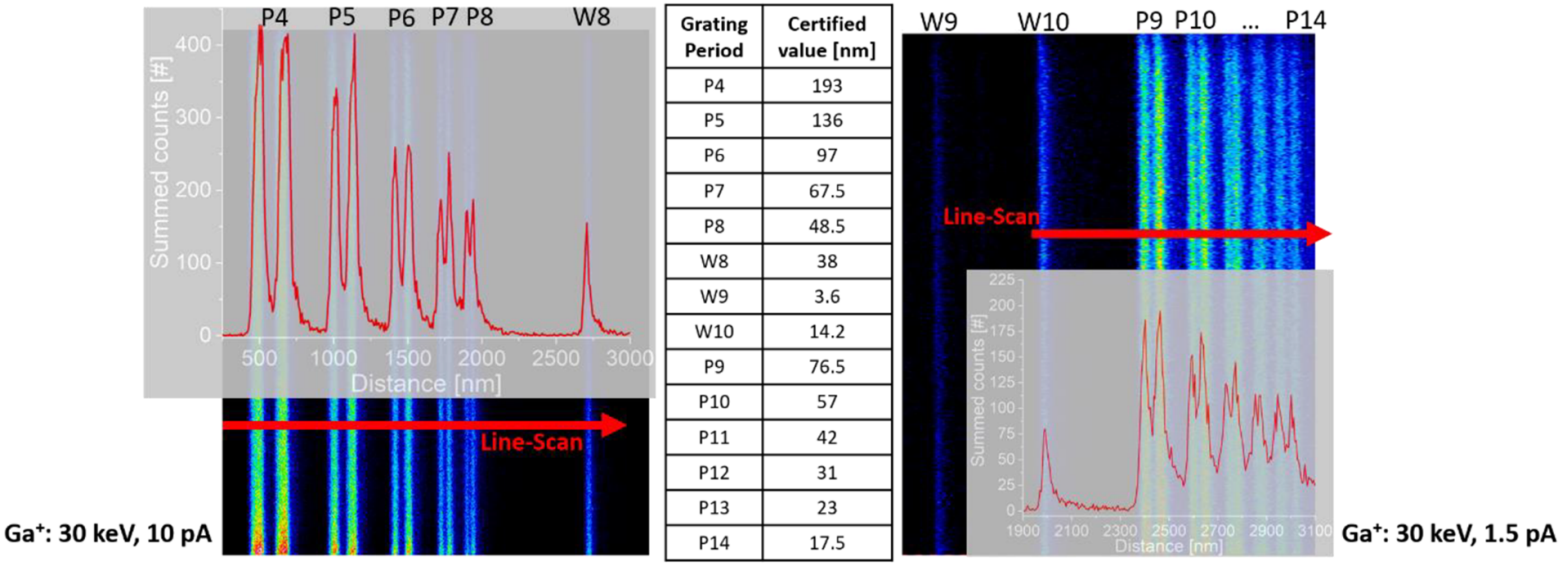
Lateral resolution determination of magnetic sector SIMS imaging on the certified reference sample BAM-L200 using the Ga-FIB on the FIB-SEM-SIMS instrument. (left) Al^+^ SIMS map of the grating period region from P4 to P8 with an overlay of a 10-pixel integrated intensity profile line-scan (Ga-FIB: 30 keV, 10 pA; cut-out of FoV 3.5 × 3.5 μm^2^ imaged with 512 × 512 pixels (6.8 nm/pixel); dwell time of 0.5 ms). (center) Certified values of grating period and single line sizes.^24^ (right) Al^+^ SIMS map of the grating period region from P9 to P14 with an overlay of a 10-pixel integrated intensity profile line-scan (Ga-FIB: 30 keV, 1.5 pA; cut-out of FoV 5.2 × 5.2 μm^2^ imaged with 1024 × 1024 pixels (5.1 nm/pixel); dwell time of 0.5 ms).

SIMS imaging is one of the main interests in coupling a mass spectrometer to an FIB instrument. The SIMS imaging resolution was evaluated using a standard BAM-L200 sample;^24,30^ see Figure 5. A 30 keV Ga^+^ beam was used in two different current settings to image two different grating period parts of the sample. Prior to the SIMS imaging, the sample was cleaned over a larger field of view (FoV) using the Ga^+^ beam (30 keV, 150 pA) to remove any surface contamination until the aluminum lines were clearly seen in the SE imaging mode.

The left-hand side of Figure 5 shows the Al^+^ signal image obtained with a Ga^+^ beam setting of 30 keV and 10 pA, scanned over an FoV of 3.5 × 3.5 μm^2^ (only part of the FoV is shown) representing the grating period region from P4 to P8. The scanning was performed at 512 × 512 pixels (6.8 nm/pixel) with a dwell time of 0.5 ms/pixel. Further shown is the intensity profile obtained across the periods in which the counts from a 10-pixel large line scan were integrated, as indicated in the image. All periods down to P8 (48.5 nm) were resolved, and even the single line W8 (38 nm) can be seen. When performing the rising-edge resolution evaluation at the imaged grating periods, as was also done on the same sample by Kollmer et al.^31^ and Kim et al.,^32^ a lateral resolution of 15 nm can be determined in SIMS imaging mode (80%–20% maximum intensity drop criterion was used as proposed in ref (31) here across the rising edges of P5 and P6). The right-hand side of Figure 5 shows the second grating period region, namely, P9 down to P14, this time using a 30 keV, 1.5 pA Ga^+^ beam. It represents again a cutout of an FoV of 5.2 × 5.2 μm^2^, taken with 1024 × 1024 pixels (5.1 nm/pixel) and a dwell time of 0.5 ms/pixel. When one considers the line scan, the period down to P12 (31 nm) is resolved, and P13 (23 nm) is still partially resolved. Here, even a faint signal from line W9 (3.6 nm) and a more pronounced signal from line W10 (14.2 nm) can be seen. Performing again the rising-edge resolution determination at the periods P9 and P10, a lateral resolution value of 15 nm is confirmed. These images were obtained with a careful choice of FoV size, pixel matrix size, and counting time per pixel for the different beam current settings used, as the sample, and especially the Al lines, were very easily degraded with an incorrect acquisition setting.

### SIMS Performance: 3D SIMS Volumetric Reconstruction

To demonstrate the instrument’s performances in terms of spatial and depth resolution in a single representation, a 3D volume reconstruction was created using the SIMS image frames acquired from a patterned cuboid structure presented in the Supporting Information (Figure S3).

**Figure 6:**
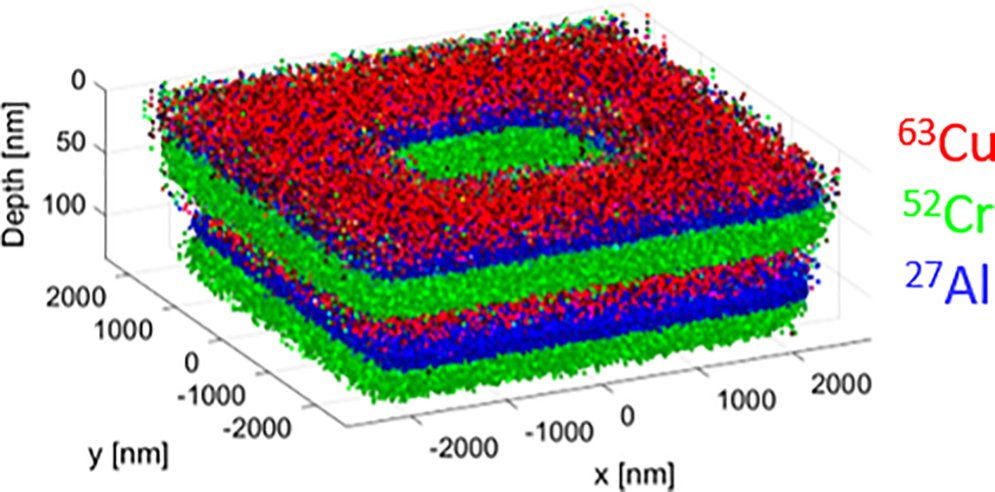
Side view of the 3D SIMS volume reconstruction of a multilayered cuboid structure using 180 SIMS image planes corrected for the thickness of each layer.

Thus, the RGB images containing the acquired ^63^Cu (red), ^52^Cr (green), and ^27^Al (blue) signals were aligned and converted into a point cloud in a 3D space in MATLAB. The correct thickness of each layer was attributed to the corresponding volume in the point cloud. Figure 6 shows the reconstructed cuboid in a side view. With this 3D reconstruction, we demonstrate the capability of the Scios-SIMS to create full 3D volume reconstructions of specimens consisting of different compositions.

### Applications in Materials Science and Life Sciences

The novel FIB-SEM-SIMS instrument has been applied to different topics in order to address scientific questions and to demonstrate the instrument’s strong analytical potential. Here we will focus on applications in the field of materials science for energy conversion and storage, a new alloy for aerospace applications, and an overview of the potential of the FIB-SEM-SIMS in the field of life sciences.

### Solar Cells

**Figure 7:**
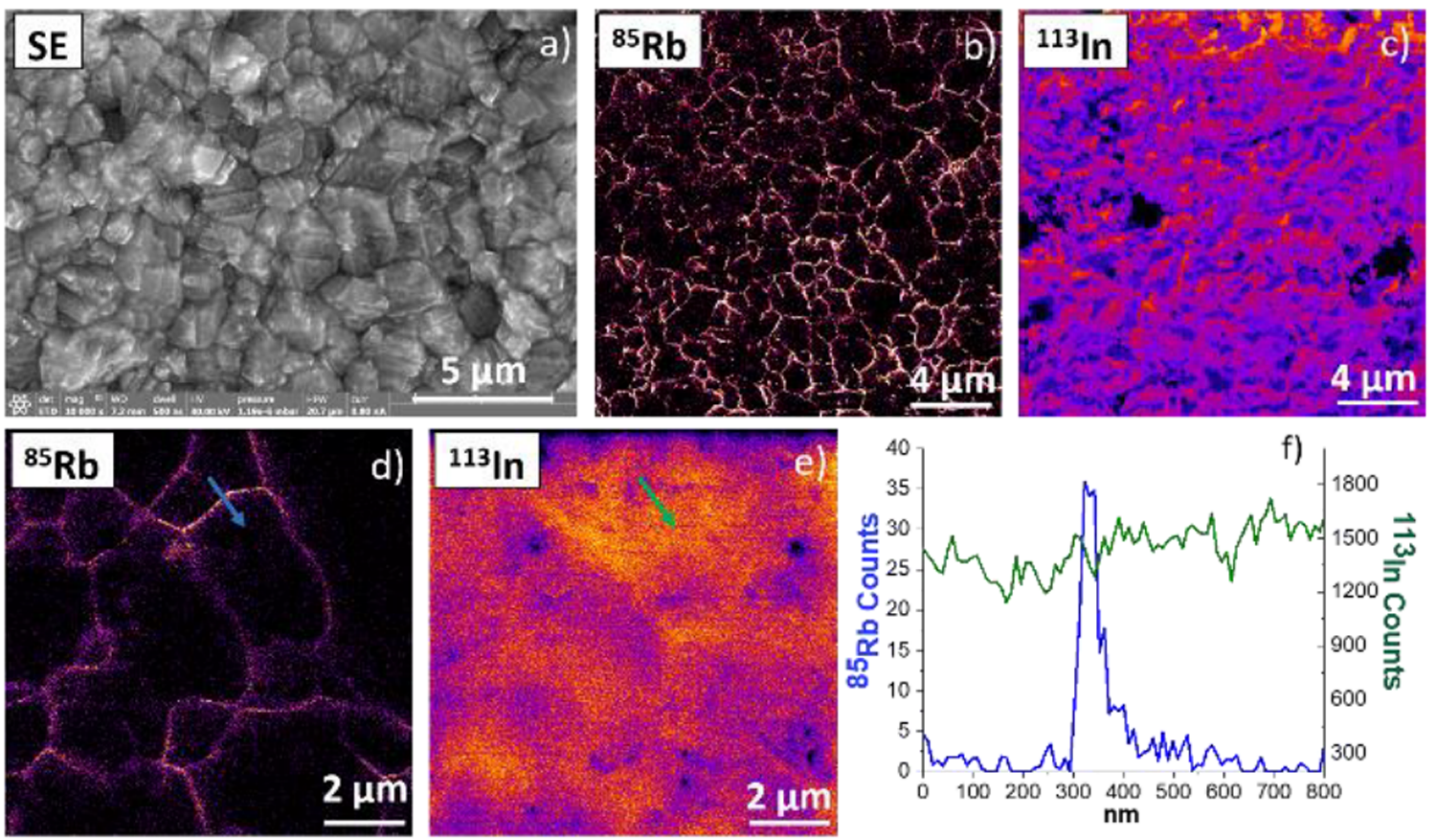
CIGS solar cell after a rubidium fluoride (RbF) postdeposition treatment was applied. (a) SE image (1536 × 1092 pixels; electron beam at 30 keV, 0.8 nA); (b–e) SIMS images (512 × 512 pixels, dwell time per pixel of 5 ms; Ga^+^ beam at 30 keV, 3 pA; FoV (b, c) 20 × 20 μm^2^/(d, e) 5 × 5 μm^2^); (f) line profiles (10 pixels in width as indicated by arrows in (d, e)), showing that rubidium is accumulated only in the grain boundary.

In recent years, for photovoltaic conversion, many studies have been undertaken to allow a significant increase in the conversion efficiency as well as the stability of such devices with time. For copper indium gallium selenide solar cells, it has been demonstrated that treatments during the elaboration of CIGS thin films could allow one to obtain higher yields. An alkali fluoride (e.g., RbF) postdeposition treatment is applied after the growth of the CIGS layer, on the order of 1 wt %, allowing one to obtain a solar cell efficiency of 22.6%.^25^ The investigation of the rubidium distribution in the grains and their boundaries requires high lateral resolution and high sensitivity. The localization determination of the alkali metal was previously performed by Atom Probe Tomography (APT) and high-resolution synchrotron-based X-ray fluorescence analysis (nano-XRF). In the first case, APT requires a complex sample preparation, without allowing the entire film to be analyzed, although it provides atomic resolution. In the second case, nano-XRF is a quantitative technique but with a limited spatial resolution (few μm). In these previous studies, rubidium was detected only in the grain boundaries, with a detection limit of 200 ppm. Using the FIB-SEM-SIMS instrument, we were able to detect Rb in the grain boundaries (Figure 7) with a detection limit that is 10 times better than with the synchrotron technique.^25^ Furthermore, with SIMS, we confirmed the absence of Rb inside the grains after we performed acquisitions on various ROIs (from 20 × 20 μm^2^ to 5 × 5 μm^2^) analyzed in just a few minutes (20 min per ROI using a pixel size of 12 nm), without complex sample preparation.

### Batteries

The prominence of lithium ion batteries for mobile and stationary use requires the improvement of their performance to meet increasingly demanding requirements for energy storage, by the optimization of suitable electrodes and electrolytes.

**Figure 8:**
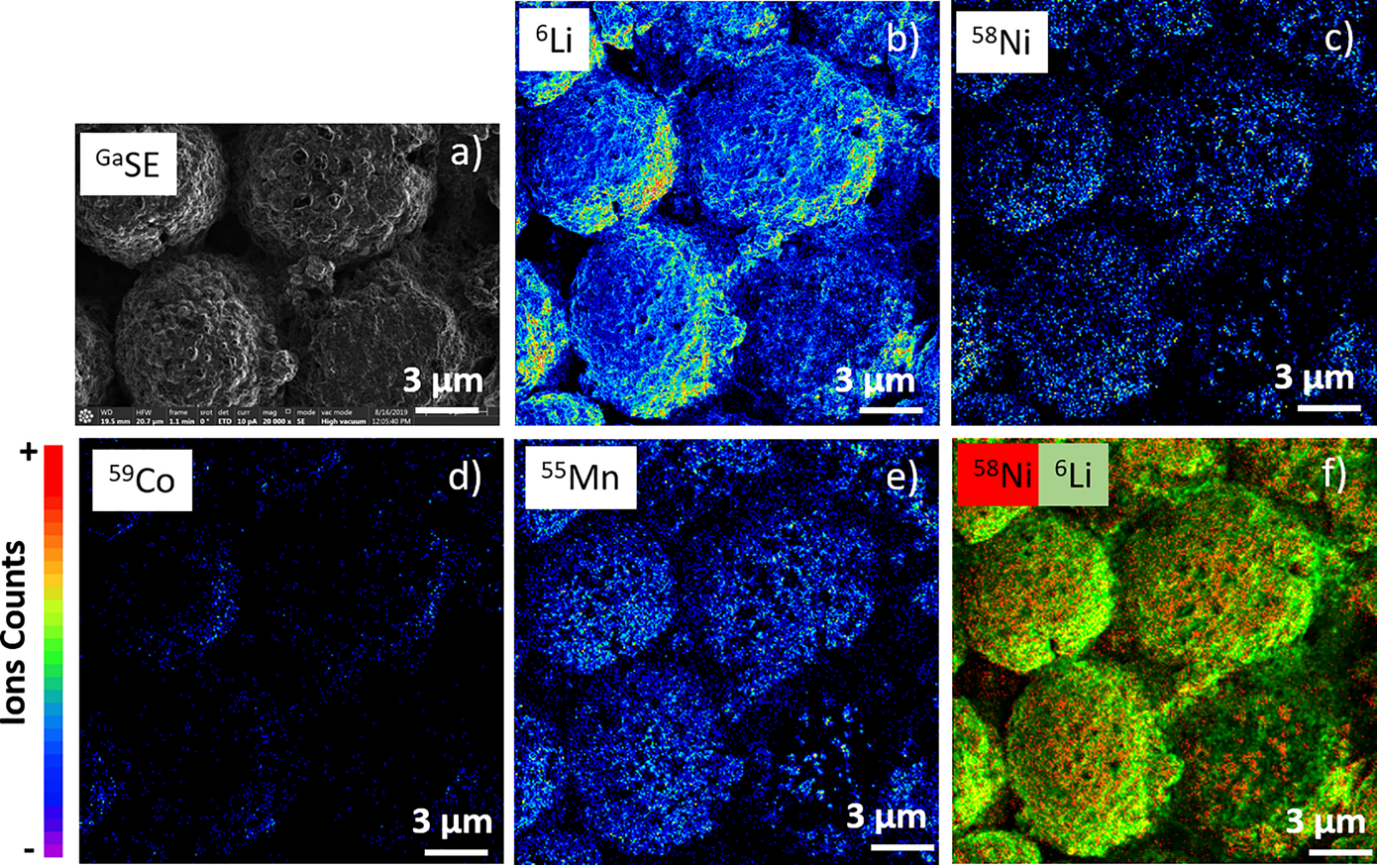
LiNiCoMn cathode material for Li ion batteries. (a) SE image (1536 × 1092 pixels, dwell time per pixel 5 μs; Ga^+^ beam 30 keV, 10 pA); (b–e) SIMS images (FoV 20 × 20 μm^2^, 512 × 512 pixels, dwell time per pixel 2.5 ms; Ga^+^ beam 30 keV, 3 pA). The color intensity scale is from 0 to 20 (black to red). (f) The image represents an overlay of lithium (green) and nickel (red).

Unlike analytical techniques based on X-rays, that is, EDX, SIMS allows the detection of lithium at a very low detection limit, together with alkali and alkaline earth elements. To investigate degradation at the interfaces of batteries, which is reducing the storage performance, a cycled LiMnNiCo battery cathode was extracted from disassembled cells and analyzed on the FIB-SEM-SIMS (Figure 8). The side previously in contact with the electrolyte was imaged to observe both morphological and chemical modifications. The elements recorded were Li, Mn, Co, and Ni. The major isotopes were selected, except for lithium, for which the ^6^Li isotope (7.5% abundance) was chosen in order not to saturate the corresponding detector. In this study, we can see the effect of the degradation of the cathode previously in contact with the electrolyte. On top of a granular morphology, while manganese is almost homogeneous, nickel and cobalt present a more heterogeneous distribution. Various hypotheses related to the degradation of the cathode can be listed. The one that seems most likely is a diffusion of nickel and cobalt in the electrolyte to form a solid electrolyte interphase.

To verify this, it would be necessary to analyze the whole cell in section, that is, the complete cathode/electrolyte/anode system. One of the advantages of the FIB-SEM system is that it is possible to do an in situ analysis, minimizing the risk of contamination (high reactivity of Li with air) and allowing us to obtain the flattest possible analysis surface.^33^ We note that the samples can be also prepared in a glovebox and transferred to the instrument using an inert gas atmosphere shuttle. Recently, our FIB-SEM-SIMS instrument was equipped with a load-lock for rapid sample introduction and docking station for the shuttle (Ferrovac, CH).

### Alloys

Thanks to their mechanical performance and lightness, aluminum–lithium (Al–Li) alloys are extensively used for applications in the aerospace industry.

The latest Al–Li alloys contain, in particular, copper, magnesium, manganese, and zinc as elements, in the range of 0.2–2 wt %. The microstructure of these materials typically includes finely distributed nanoscale-strengthening precipitates. The size and distribution of these precipitates have a significant impact on the mechanical performance of these alloys. However, the analysis of Li and the low concentration of the inclusions is challenging, as many techniques, such as EDX, have low elemental sensitivity and/or insufficient spatial resolution (Supporting Information).

**Figure 9:**
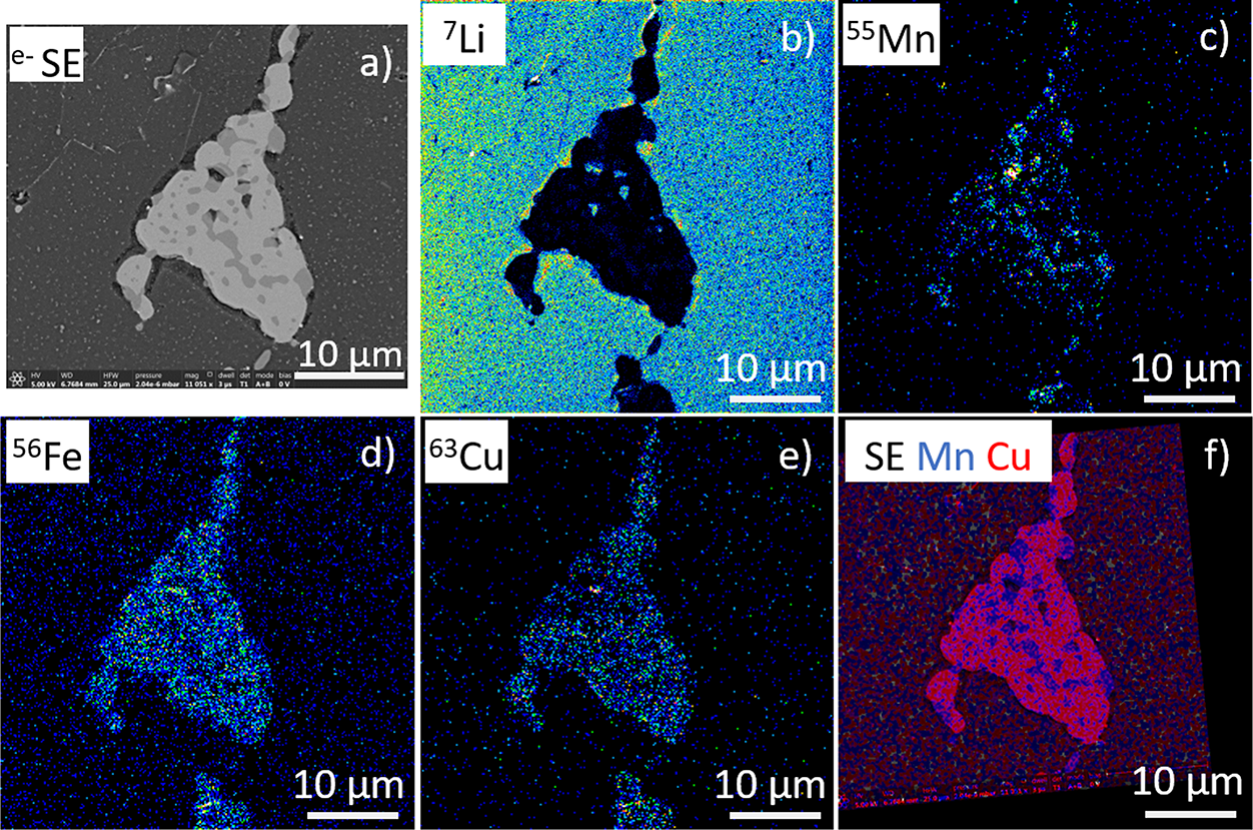
Al–Li alloys. (a) SE image (1536 × 1092 pixels; electron beam of 5 keV, 10 pA); (b–e) SIMS images (FoV 25 × 25 μm^2^, 512 × 512 pixels, dwell time per pixel of 2.5 ms; Ga^+^ beam of 30 keV, 10 pA); (f) IHS image fusion^34^ of the SE image (shown in (a)) with an overlay of the two SIMS images of (c) and (e).

First, the same ROI previously analyzed by EDX, on another dual-beam instrument (Helios, Thermo Fisher), was localized and imaged in SEM mode at normal incidence (Figure 9a). This acquisition was done while the SIMS extraction system was retracted, allowing a quick approach to search for ROIs. Second, the SIMS extraction optics was inserted, and ^7^Li, ^55^ Mn, ^56^Fe, and ^63^Cu ions were mapped in SIMS mode on the same ROI (Figure 9b–e).

The observations of intermetallic phases and the main phase are in accordance with the previous results.^26^ An FIB-SIMS analysis reveals segregation of Li at grain boundaries, which can be confirmed by BSE data.^26^ The identification of the different phases (Al_2_CuLi, T1), as described by Xu et al.,^26^ is represented in the Supporting Information Figure S4. Magnesium was detected in high-resolution images, despite its low concentration (0.2%). Xu et al.^26^ also estimate the grain boundaries to have a width of ∼90 nm. The composition of the main phase is much better spatially resolved in the FIB-SIMS results here than in EDX, showing that they are not homogeneous in composition but, rather, are made up of nanodomains (Figure 9c–e). To further push the data treatment, SE and SIMS images were correlated; that is, the raw intensities from SIMS images were combined with the SE image to form a new image. To do this, a series of corresponding landmarks was identified in both images. These were used to calculate the parameters for an affine transformation, applicable either to the SE or to the SIMS images. This overlay can be done by the addition and transparency of the images or by data pixel fusion. In this context, Vollnhals et al.^34^ proposed different SE-sharpening methods (Laplace fusion, Intensity–Hue–Saturation) to provide a high-quality correlative SE-SIMS image. The image presented in Figure 9f is obtained by the Intensity-Hue-Saturation (IHS or HIS) sharpening technique operating at the level of spatial frequencies and by including the local information present in the high-resolution (SE) image into the lower-resolution one (SIMS). In this study, the registered SIMS images were fused with the SE image, using ImageJ and red (copper) and blue (manganese) colormaps applied to the different SIMS data sets. Hence, the nanograins are better resolved with respect to the signal dynamics obtained by EDX (Supporting Information Figure S5) and are thus more helpful for the data interpretation.

### Life Sciences

The FIB-SEM-SIMS enables one to do an in situ investigation of both the morphology and the chemical composition of biological samples, such as tissues and cells. This is particularly interesting in toxicology and pharmacology. For example, metal nanoparticles, halogenated pollutants, metal-based drugs, or halogenated drugs can be localized at cellular and subcellular levels. Alternatively, molecules of interest labeled with a metal, halogen, or less abundant isotope can be traced by detecting the label. Consequently, the mechanisms of action of these chemicals, and therefore their potential effect on the body, can be investigated.

Halogenated persistent organic pollutants, such as brominated flame retardants or fluorinated substances, are toxic chemicals related to many human diseases. Given its sensitivity and spatial resolution, FIB-SEM-SIMS is perfectly suited to assessing the fate of these pollutants.

**Figure 10:**
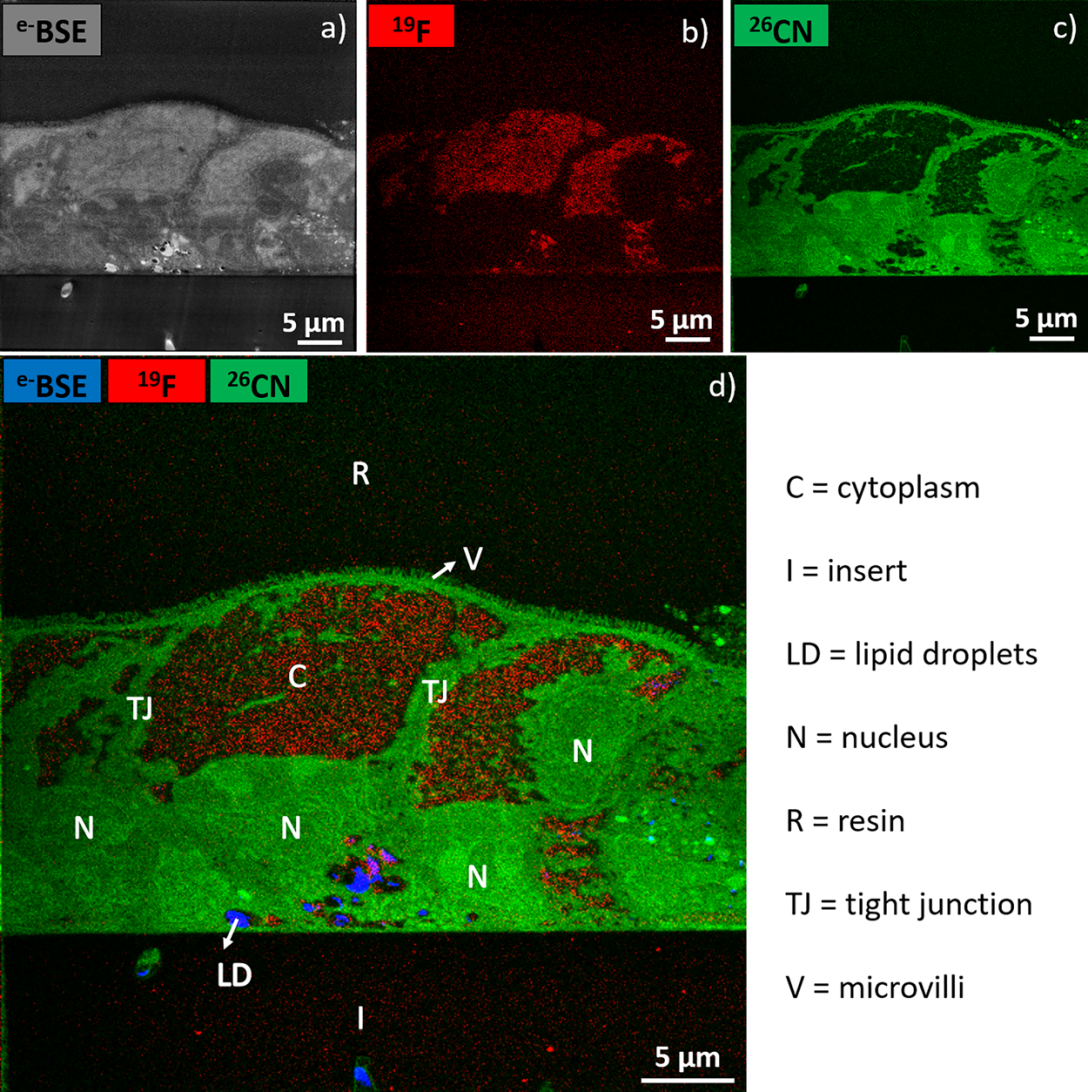
Intenstinal cells exposed to fluorinated toxicant: (a) BSE image (FoV of 70 × 70 μm^2^, 6144 × 4096 pixels, dwell time per pixel 5 μs; electron beam 5 keV, 0.2 nA); (b, c) SIMS images (FoV of 40 × 40 μm^2^, 512 × 512 pixels, dwell time per pixel 4 ms; Ga^+^ beam 30 keV, 0.1 nA). (d) Overlay image.

The BSE image in Figure 10a displays a cellular contrast, well-known in biological microscopy,^35^ due to contrast agent staining. The SIMS images in Figure 10b,c enable us to localize a fluorinated environmental pollutant (in red) and the cells (in green). The fused image in Figure 10d provides information about the uptake and localization of the pollutant inside the cells. As the compound is mainly localized in the cytoplasm of the cells, we could assume it has an impact on their cytosol functions, such as signal transduction between the cell membrane and the nucleus and organelles, metabolite transport activities, cytokinesis, and metabolism. Obviously, complementary toxicity tests are necessary to evaluate the toxicology of this compound and the possible mechanisms of action. However, SIMS imaging reveals useful information about localization from tissular to subcellular levels that none of the usual toxicity tests could provide, which makes it very attractive.

One critical step for SIMS imaging of biological samples is their preparation. As they are investigated under vacuum conditions, common protocols for electron microscopy are usually applied. As for electron microscopy, biological specimens should be processed in a way that, when analyzed, they resemble their original living state as much as possible. However, because of the numerous processing steps involved, such as fixation, dehydration, and embedding, chemical information might be altered (washout and/or redistribution) when the molecules of interest are not reactive toward fixatives. Thus, the interpretation of SIMS images requires the careful attention of the investigator. In some cases, cryo-preservation can be favored over traditional resin embedding, but this will undoubtedly induce other challenges. One advantage in life science is that thin sections are usually analyzed, which overcome topographic issues encountered for other applications in SIMS.

## Conclusion

A magnetic-sector double-focusing SIMS system was developed and successfully integrated into a dual-beam instrument as an add-on analytical tool. In the chosen configuration, the SIMS extraction optics are retractable and are inserted for SIMS operation directly above the sample surface, the latter one being at the FIB-SEM instrument’s eucentric point. This configuration allows for highest secondary ion extraction efficiencies and, hence, the highest sensitivity, while being able to access the region of interest in the sample with both the ion and the electron beam. The FIB-SEM-SIMS system can be used to generate mass spectra, 2D images, 3D images, and depth profiles. Mass spectra can be recorded on a mass range from 1 to 400 amu with a mass resolving power of above 400 and high dynamic range, while allowing isotopic selectivity. The SIMS imaging results demonstrated the possibility of recording high-resolution chemical maps with a lateral resolution of 15 nm. These SIMS images can be stacked into a 3D volume reconstruction, giving a full representation of the analyzed volume. When using the FIB-SEM-SIMS for depth profiling, a good depth resolution can be achieved when lowering the landing energy of the Ga^+^ beam. A depth resolution of ∼4 nm was demonstrated at 3 keV on a multilayer sample.

Different materials science application results have demonstrated that this newly developed FIB-SEM-SIMS system can be used for high-resolution high-sensitivity correlative investigations in current topics related to energy storage and energy production devices (i.e., batteries, solar cells). Moreover, the possibility of performing 3D volume SIMS imaging with an excellent lateral and depth resolution opens interesting prospects for investigating complex 3D architectures, such as in microelectronic devices. Beyond materials science, the instrument can further be used to address scientific questions in earth or life sciences.

To summarize, installing a high-performance magnetic sector SIMS system on FIB-SEM instruments offers significant added value for a large variety of applications, including highly sensitive analytics, highest-resolution SIMS imaging (resolution of 15 nm) and depth profiling (depth resolution of a few nm), in situ process control during patterning/milling, and direct correlation of SIMS data with other analytical or imaging data obtained on the same instrument, such as high-resolution SE images or EDX spectra/data.

## Supporting Information Available

Photograph of FIB-SEM-SIMS instrument; Sc-Ultra depth profiling data of Cu/Cr/Al multilayer; SE image and depth profiles of multilayer cuboid structure; FIB-SIMS images of Al–Li alloys and SEM-EDX data of Al–Li alloys (PDF)
