# Supplementary material for: Magnetic Sector Secondary Ion Mass Spectrometry on FIB-SEM Instruments for Nanoscale Chemical Imaging
Source: Anal Chem. 2022 Jul 21;94(30):10754–63. doi: 10.1021/acs.analchem.2c01410 (PMC9352148; doi:10.1021/acs.analchem.2c01410)
Supplement: Supplementary file 1 — ac2c01410_si_001.pdf [file ac2c01410_si_001.pdf]

## Supporting Information:

# Magnetic sector secondary ion mass spectrometry on FIB-SEM instruments for nanoscale chemical imaging

*Olivier De Castro*[*1], Jean-Nicolas Audinot[1], Hung Quang Hoang[1], Chérif Coulbary[1], Olivier Bouton[2], Rachid Barrahma[2], Alexander Ost[1,3], Charlotte Stoffels[1,3], Chengge Jiao[4], Mikhail Dutka[4], Michal Geryk[5] and Tom Wirtz[1]*

*[1]Advanced Instrumentation for Nano-Analytics (AINA), MRT Department, Luxembourg Institute of Science and Technology, 41 rue du Brill, L-4422 Belvaux, Luxembourg;*

*[2]Prototyping, MRT Department, Luxembourg Institute of Science and Technology, 41 rue du Brill, L-4422 Belvaux, Luxembourg;*

*[3]Faculty of Science, Technology and Medicine, University of Luxembourg, 2 Avenue de l'Université, 4365 Esch-sur-Alzette, Luxembourg.*

*[4]Thermo Fisher Scientific, Achtseweg Noord 5, 5651 GG, Eindhoven, Netherlands.*

*[5]Thermo Fisher Scientific, Vlastimila Pecha 12, 627 00 Brno, Czech Republic.*

**corresponding author: olivier.decastro@list.lu*

# Table of Contents

**Photograph of FIB-SEM-SIMS prototype instrument** ........ **S2**

**Sc-Ultra SIMS data of Cu/Al/Cr multilayer sample** ........ **S2**

**SE image and depth profiles of multilayer cuboid structure** ........ **S3**

**FIB-SIMS data of Al-Li alloy sample** ........ **S3**

**SEM-EDX data of Al-Li alloy sample** ........ **S4**

## Instrument design

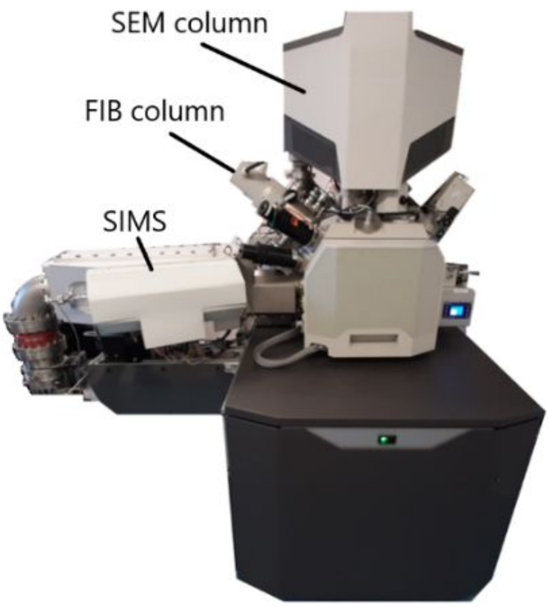


**Figure S1**: Photograph of FIB-SEM-SIMS prototype instrument.

## SIMS performance: Depth profiling + 3D imaging

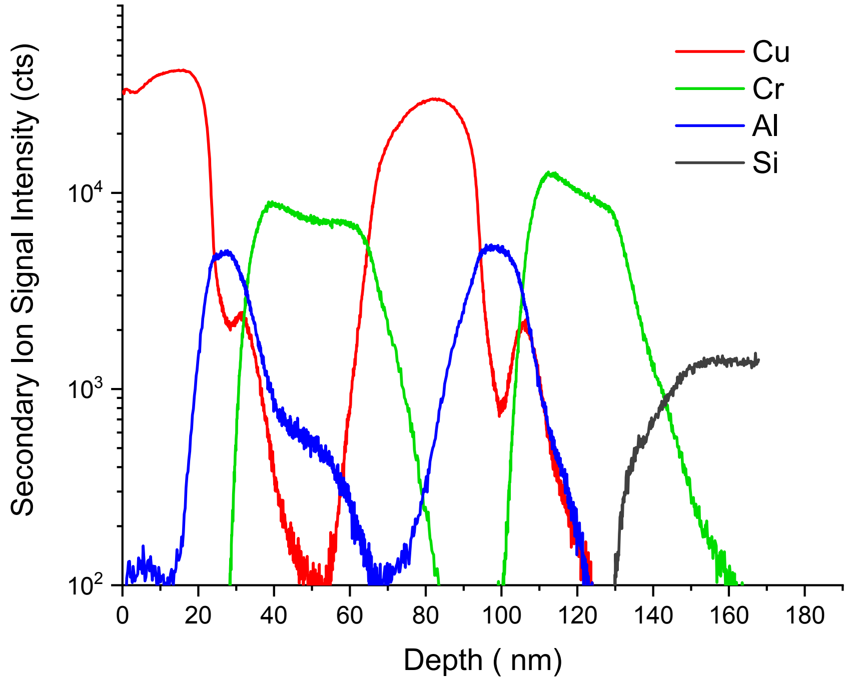


**Figure S2**: Sc-Ultra SIMS depth profile of the Cu/Cr/Al multilayer. Analysis of Cu (23nm)/Al (11nm)/Cr (34nm)/Cu (27nm)/Al (14nm)/Cr (27nm)/Silicon substrate was performed with an impact energy of 1 keV with a $Cs^+$ primary ion beam. The successive layers are visible in the SIMS depth profile. The

thickness of the layers was determined by profilometry. The sputtering rates of the layers of Al, Cu and Cr were $3.3 \cdot 10^{-3}$ nm/s, $1.3 \cdot 10^{-2}$ nm/s and $4.4 \cdot 10^{-3}$ nm/s, respectively.

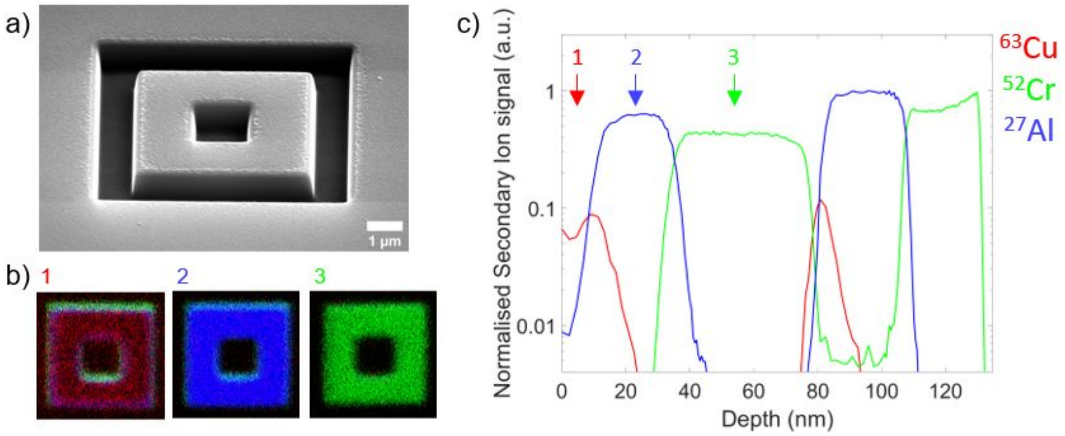


**Figure S3:** a) SE image of a milled cuboid structure for depth profiling and 3D imaging purposes. The cuboid has been patterned by FIB milling at 30 keV beam landing energy and 0.5 nA primary $Ga^+$ current. Its surface area is 5x5 µm$^2$ with a central crater (hole) of 1.85x1.85 µm$^2$ surface area; b) SIMS acquisitions of the cuboid at different acquisition times: after 12 min. (left), 1 hr. (center), 4 hr. 18 min. (right). RGB colors represent the ion species analyzed (red: $^{63}Cu$, green: $^{52}Cr$, blue $^{27}Al$); c) Depth profile created by integrating the counts for each element obtained in each image frame (in arbitrary units). Normalization performed with respect to the $^{69}Ga$ signal measured in parallel to correct for instrumental drifts of the beam alignment due to the long acquisition time. The arrows and numbers indicate the depth at which the images in b) were acquired.

## Applications: Alloys

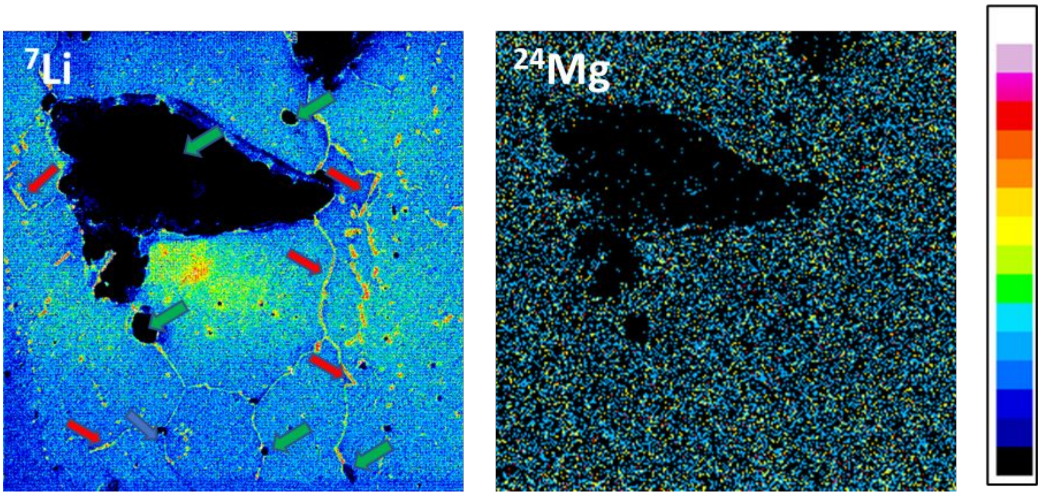


**Figure S4:** FIB-SIMS chemical maps of Al-Li alloys showing the distribution of $^{7}Li^+$ and $^{24}Mg^+$. The arrows indicate the secondary phases present in the area of interest, in red the phase T1 ($Al_2CuLi$) and in green the intermetallic phase (without lithium)[1]. The colour scale is presented based on counts per pixel of the chemical maps (0 to 150 for $^{7}Li^+$ and 0 to 5 for $^{24}Mg^+$). Field-of-view of 40 x40 µm$^2$. Ga 30keV, 5 pA, 512 x 512 pixels.

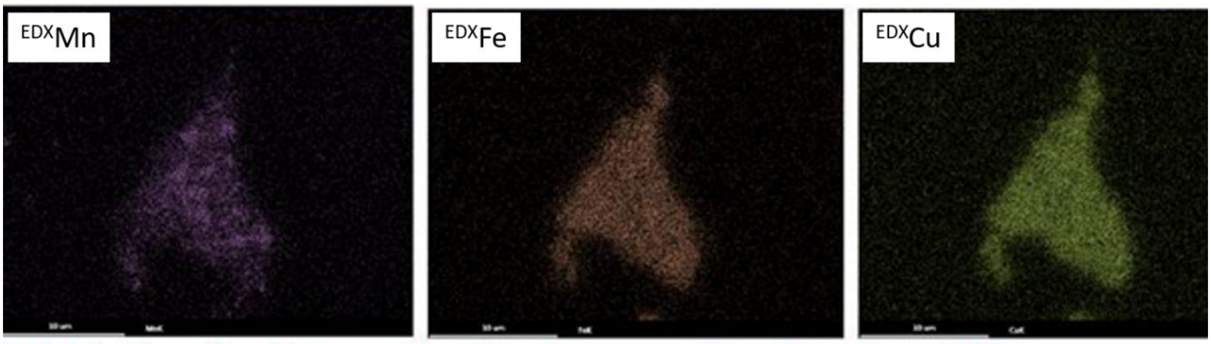


**Figure S5:** SEM-EDX maps of Al-Li alloys showing manganese, iron and copper distributions. Lithium and magnesium were not detected by EDX. The lack of both spatial resolution and sensitivity of EDX does not allow to resolve the grain heterogeneity.

**References:**

(1) Xu, X.; Jiao, C.; Li, K.; Hao, M.; Moore, K. L.; Burnett, T. L.; Zhou, X. Application of High-Spatial-Resolution Secondary Ion Mass Spectrometry for Nanoscale Chemical Mapping of Lithium in an Al-Li Alloy. *Mater. Charact.* **2021**, *181*, 111442.
